# Estimating of the costs of nonfatal occupational injuries and illnesses in agricultural works in Thailand

**DOI:** 10.1057/s41271-020-00251-0

**Published:** 2020-09-07

**Authors:** Densak Yogyorn, Craig Slatin, Somkiat Siriruttanapruk, Susan Woskie, Thanawadee Chantian, Pusanisa Chaladlerd, Pornpimol Kongtip

**Affiliations:** 1 Department of Occupational Health and Safety, Faculty of Public Health, Mahidol University, Bangkok, 10400 Thailand; 2 Department of Public Health, University of Massachusetts Lowell, Lowell, MA USA; 3 Bureau of Occupational and Environmental Disease, Ministry of Public Health, Nonthaburi, Thailand

**Keywords:** Agricultural, Occupational injury and illness (OII), Costs

## Abstract

Thailand lacks occupational injury and illness (OII) surveillance for its agricultural sector, a sector that comprises 34% of the total Thai workforce but is not covered by the workers compensation system. This study used data from Thailand’s Universal Health Care System to estimate the medical costs of OIIs from agricultural work in Thailand. In 2017, OII medical costs totaled $47 million (USD), about ~ 0.2% of the gross domestic product produced by the Thai agricultural sector. We recommend that some of the national funds currently used for medical treatment of OIIs be used instead to develop and implement prevention programs in agriculture. This would improve not only worker health and safety, but also productivity. Availability of data on working conditions, injuries and illnesses, and especially lost time, lost income and productivity, and OII-related costs for the workers and their dependents might enable better public health policy formulation.

## Introduction

In recent years, the importance of Thailand’s agricultural sector to the national GDP has declined from 10.5% in 2014 to 8.6% in 2018 [1]. Agriculture remains part of Thailand’s economic growth, having expanded by 5.0% in 2018, and employing over 13 million workers (34%) [2] in 2018. These agricultural workers constitute 40% of those below the poverty line (~ $3 per day) in Thailand [3].

Thailand’s Labor Protection Act covers workers in the private sector but does not apply to informal sector agricultural workers [4]. Agricultural workers are exposed to physical hazards such as heat stress due to the equatorial climate, biological hazards such as insect-borne diseases and parasites, chemical hazards such as pesticides and herbicides [5]. They also face safety hazards due to the use of hand tools, poorly guarded machinery, repetitive and awkward postures, and inadequate use of personal protective equipment. These common hazards, present in the many and frequent agricultural tasks, put farmers at risk of occupational injuries and illnesses [6]. Farmers often seek medical treatment at local primary healthcare units (PCUs), also called health-promoting hospitals, or at district or provincial hospitals for more intensive levels of care.

Thailand’s Universal Health Coverage Scheme (UHCS) is a tax-financed scheme that provides free healthcare at the point of service introduced in 2002 [7]. The UHCS covers approximately 47 million people, 75% of the country’s population. These are mostly low-wage informal sector workers, as formal sector employees are covered under the 1990 Social Security Act. As the main social health insurance scheme in Thailand, the UHCS accounts for 17% of the country’s total health expenditures. Informal agricultural workers who live in rural areas with local healthcare services constitute the majority of UHCS’s beneficiaries [8]. The beneficiaries receive services almost identical to those provided to formal sector workers through the national Social Security Administration (SSA), including outpatient, inpatient, accident, and emergency services, plus dental care.

Formal sector workers in the SSA are also covered by the Workers Compensation Fund Act of 1994 that compensates employees who experience work-related injuries or diseases, disabilities, or die. The Workers Compensation program is the principal collector of data on occupational injuries and illnesses in Thailand. Workers Compensation does not, however, cover informal sector workers, and lacks a mechanism to subsidize informal sector worker enrollment in the SSA or Workers Compensation. These gaps and the general lack of government regulation of occupational safety and health in the informal sector have shifted medical treatment expenditures for OIIs to the UHCS [9].

The National Health Security Office (NHSO) developed a claims and reimbursement management system for the UHCS. All outpatient and inpatient medical service data from all healthcare facilities nationwide are consolidated in a health data center. To control treatment costs, UHCS employs ‘closed-end provider payment mechanisms’. That is, for outpatient services, all hospitals and PCUs are paid a flat rate for each beneficiary, capped at $39 USD per beneficiary [10]. Inpatient services are reimbursed based on the patients’ diagnosis classification using the World Health Organization’s International Classification of Diseases version 10 (ICD 10) and ICD 9-CM (Clinical modified version) [11], co-morbidities of each patient, and their treatment/length of hospital stay. The result is a Diagnosis-Related Group (DRG) code. A DRG code consists of five digits that represent Major Diagnostic Category (MDC), Disease Cluster (DC), Complication and Comorbidity (CC) [12]. The DRG-based payment system encourages hospitals and primary healthcare units to manage expenditures for purchasing drugs, supplies, and other services so as not to exceed the DRG allotment and allows comparisons in expenditures across hospitals [13]. Each provincial healthcare office collects individual outpatient and inpatient medical service data from all health service facilities within its province. These are then consolidated at the provincial data center and submitted to the centralized NHSO Health Data Center [14].

### Existing models for estimating cost of occupational illness and injury (OII)

In high resource countries with robust data systems, researchers have used workers compensation and social security disability records to estimate the cost of OIIs by multiplying the number of injuries by the average the cost of these injuries, including lost work time, to estimate the average cost [15]. In the United States (U.S.), Leigh et al. [16] were able to draw upon the data from the U.S. Bureau of Labor Statistics Survey of Occupational Injuries and Illnesses (SOII), adjusted by the United States National Institute for Occupational Safety and Health (NIOSH) fatality data. They estimated a cost of $4.57 billion as the consequence of 841 deaths and 512,539 nonfatal injuries and occupational illnesses in the agricultural sector. Even with the extensive data sources in the U.S., underestimates in the true costs of OIIs persist. Leigh et al. [17] reviewed the SOII data, the Quarterly Census of Employment and Wages, and the Current Population Survey to estimate the undercount of OIIs. Their study found that the SOII missed 73.7% of crop farm cases and 81.9% of animal farm cases. This is an important gap, reflecting the difficulty of collecting OII data for the agricultural sector even in a high resource country.

We calculated the medical costs of treating job-related injuries and illnesses among informal sector agricultural workers covered by the UHCS system. (We report all costs in US dollars.) We estimate the expenditures shifted to the UHCS system, so that policy makers can consider re-allocation of funds from medical treatment to OII prevention programs. Our calculations do not include measures of short term and disability losses in worktime, income, and production that affect the contribution of agriculture to the national GDP, as there are currently no data available to support such estimates. In addition, we propose new data collection methods that are needed to better estimate the true full cost of OIIs in the agricultural sector.

## Methods

### Data collection

At each healthcare visit the following information must be recorded: name, age, sex, marriage status, current and previous occupation, religion, education, family status, blood type, health insurance privileges, ICD-10 diagnosis code, and the medical treatment according to the ICD 9-CM (Clinical modified version). Occupation is coded using the International Standard Classification of Occupations code (ISCO) [18] and entered with other demographic information into the health data system. ISCO codes considered “agricultural work” for this study included the 3-digit codes for: Market gardeners and crop growers (611), Animal producers (612), Forestry, Fishery and Aquaculture workers (621), and Subsistence Crop farmers (631). These were then sub-categorized by 4-digit codes (Table 1). The diagnosis and medical treatment codes assigned by physicians using the standardized Diagnosis-Related Groups (DRG) were applied to calculate the reimbursable average expenditure for that patient’s treatment [19].

**Table 1:** The direct medical costs of occupational injuries in Thai agriculture (2017)

ISCO code	Occupation	Number of visits	Cost ($ USD)	Cost per visit ($ USD)
611	Market gardeners and crop growers			
6111	Field Crop and Vegetable Growers	1,453,938	24,915,162	
6112	Tree and Shrub Crop Growers	33,512	937,364	
6113	Gardeners, Horticultural and Nursery Growers	52,512	1,617,879	
6114	Mixed crop Growers	17,509	276,758	
	Subtotal for ISCO 611	1,557,471	27,747,163	18
612	Animal producers			
6121	Livestock and dairy producer	585	19,435	
6122	Poultry producers	72	599	
6123	Apiarist and Sericulturist	46	128	
6129	Animal producers not elsewhere classified	33,296	669,478	
6130	Mixed crop and animal producers	35,775	5,329,711	
	Subtotal for ISCO 612	69,774	6,019,350	86
621	Forestry, Fish and Aquaculture workers			
6210	Forestry and related workers	58	843	
6221	Aquaculture workers	607	41,421	
6222	Inland and coastal waters fishery workers	6,854	126,950	
6223	Deep-sea fishery workers	72	1457	
6224	Hunters and trappers	85	1542	
	Subtotal for ISCO 621	7676	172,213	22
631	Subsistence Crop Farmers			
6310	Subsistence crop farmers	24,436	2,567,311	
6320	Subsistence livestock farmers	809	4672	
6330	Subsistence mixed crop and livestock farmers	44,250	344,243	
6340	Subsistence fishers, hunters, trappers and gatherers	239	13,474	
	Subtotal for ISCO 631	69,734	2,929,701	42
	Total	1,704,655	36,868,427	22

*Note* Exchange rate: THB 32 = 1 US$ (Bank of Thailand)

### Method for estimating the costs of the medical treatment of an occupational injury

The data came from all facilities around the country through the UCHS and the HDC. Initially, we obtained the number of outpatient healthcare facility visits by agricultural workers (by ISCO code) who received a medical intervention identified by ICD code S00-S778 [20]. The Bureau of Occupational and Environmental Disease verified these injuries as work related. We calculated the cost of treatment based on actual expenditures incurred.

### Method used to estimate the costs of medical treatment of an occupational illness

In addition to providing diagnosis and treatment codes, the Ministry of Public Health asks clinicians to fill out a separate form (#506/2) [21] to report the diagnosis of a particular occupational disease; lung and respiratory disease, physical factor diseases (heat, noise, cold), skin diseases, musculoskeletal diseases, zoonotic and plant-caused diseases, poisoning by metals, solvents, gases, pesticides, and other chemicals. Due to the limited training and capability at local healthcare units, typically staffed by bachelors-level nurses and public health officers, we believe that the surveillance data are unreliable without review by occupational medicine specialists of the clinic data. Thus, we report data on a subset of occupational illnesses collected by the health data centers without verification by the Bureau of Occupational and Environmental Disease. We believe, based on patient reports and healthcare unit staff assessments, that these diseases are the least likely to be misclassified: musculoskeletal disorders (MSD), heat stroke, organic solvent poisoning, hearing loss, and pesticide poisoning. The treatment DRG (and therefore cost) of each job-related illness was not merged with the case diagnosis in the occupational disease surveillance data. Therefore, we assumed that the cost of any of the identified occupational illnesses of agricultural workers was equivalent to the most frequent claim (DRG 04520: respiratory infection or inflammation without significant cost and clinical complexity) [13]. Some occupational illnesses, such as hearing loss, may receive no treatment, while others, such as pesticide poisoning and musculoskeletal disorders, may receive a range of medical treatments, depending on the severity. Thus, the cost associated with all illnesses ($180 USD per visit based on DRG 04520) could be an underestimate or an overestimate in any individual case. The number of visits by agricultural workers to healthcare facilities for each identified occupational disease by ISCO was then multiplied by $180, (treatment cost of DRG 04520) to estimate the agricultural occupational illness medical treatment costs. Pesticide poisonings were an exception, as there was a particular interest by the Ministry of Public Health to collect data on DRG expenditures for pesticide poisoning, allowing a more nuanced look at these healthcare costs.

## Results

### The cost of occupational injuries in agricultural works

There were 1,704,655 occupational injuries in Thai agriculture in 2017 and $36 million US dollars in medical treatment charges. The largest number of visits was in the Market Gardeners and Crop Grower group (ISCO 611) that also made up the largest number of farmers (92%). The subgroup of Field Crop and Vegetable Growers (ISCO 6111) contributed 93% of the total visits in ISCO 611 and 85% of all injuries in agriculture. This subgroup also contributed the largest total cost across all of Thai agriculture (68%), largely because this group includes most rice farmers because rice is the most widely grown crop for domestic consumption and export [22]. In terms of per capita cost, animal producers and livestock handlers suffered more severe injuries, resulting in a higher per cost visit ($86 USD per visit) compared to $22–$42 USD per visit for other types of farming (Table 1).

### The cost of occupational illnesses

The largest number of occupational illness diagnoses came from Musculoskeletal Disorders (MSD), at 65,940 visits (81%). MSDs also represent the greatest total cost for medical treatment, $11.9 million (Table 2). Hearing loss was then the second most commonly diagnosed occupational disease but contributed only 19% of visits and medical costs (Table 2). As with injuries, the largest number of visits for occupational illnesses was in the Market Gardeners and Crop Grower group (ISCO 611) with the subgroup of Field Crop and Vegetable Growers (ISCO 6111) contributing 98% of all visits and medical treatment costs in agriculture.

**Table 2:** The direct medical costs of occupational illnesses in Thai agriculture (2017)

Agricultural occupations	# MSD visits	# Hearing loss visits	Total visits	Total cost ($ USD)
6111: Field Crop and Vegetable Growers	65,094	14,525	79,619	14,331,420
6112: Tree and Shrub Crop Growers	72	178	250	45,000
6113: Gardeners; Horticultural and Nursery Growers	12	61	73	13,140
6114: Mixed Crop Growers	75	127	202	36,360
612: Animal producers	538	7	545	98,100
621: Forestry and Related workers	69	25	94	16,920
631: Subsistence Crop Farmers	80	259	339	61,020
Total cases	65,940	15,182	81,122	14,601,960
Total cost ($US)	11,869,200	2,732,760	14,601,960	14,614,560^a^

*Note* Exchange rate: THB 32 = 1 US$ (Bank of Thailand)

^a^There were additional costs from Heat stroke and organic solvent poisoning added to the total: (i) 36 heat stroke cases which cost $6480 (32 in field crops and vegetable growers; 1 in Tree and Shrub Crop Growers, 3 in Animal Producers), (ii) 34 organic solvent poisoning cases which cost $6120 (27 in crops and vegetable growers, 1 in Mixed Crop Growers, 1 in Animal Producers, 5 in Subsistence Crop Growers)

When examining the 13,955 cases of pesticide poisoning by type of pesticide used, we found that the highest number of cases was for organophosphate and carbamate insecticides. The largest per case and total treatment cost was for medical treatment of poisoning by herbicides and fungicides at $41 per case, compared to $7–$22 for poisoning by other types of pesticides (Table 3).

**Table 3:** Types of pesticide poisoning among Thai agriculture (2017)

Code	Types of pesticide	Number of cases	Cost of medical treatment ($USD)	Cost per case ($ USD)
T600	Organophosphate and carbamate insecticides	4562	72,302	16
T601	Halogenated insecticides	438	2980	7
T602	Other insecticides	1526	10,494	7
T603	Herbicides and fungicides	2597	105,493	41
T604	Rodenticides	214	4693	22
T608	Other pesticides	1012	15,938	16
T609	Pesticide, unspecified	3606	24,491	7
	Total	13,955	236,390	17

*Note* Exchange rate: THB 32 = 1 US$ (Bank of Thailand)

### Total medical costs of occupational injury and illness (OIIs)

We estimated the total medical costs of all OIIs paid for by the Thai UCHS to be US$51 million USD, which is 0.2% of the GDP of the Thai agricultural sector ($19,709 million USD in 2017) (Table 4) [23]. In Thailand, farming is family-based agriculture that is labor intensive and minimally mechanized production of low-price commodities. If the cost of inputs such as seed, fertilizer, equipment, and pesticides were deducted from the agricultural GDP, the estimated annual income of each farmer would be even lower than the estimate of $1485 without those deductions. This estimated annual income is much lower than the overall Thai income (total GDP/Thai adult population) of $6729 [24].

**Table 4:** The Medical Costs of all Occupational Injuries and Illnesses in Thai Agriculture (2017)

Occupation and ISCO code	Total number of worker	Total number of visits	Number of total visits with injury (%)	Number of total visits with diseases (%)	Cost of injury	Cost of illness	Total cost	Cost per capita
					($ USD)	($ USD)	($ USD)	($ USD)
611: Market gardeners and crop growers	12,235,856	1,637,676	1,557,471 (95.1)	80,205 (4.9)	27,747,163	14,436,900	42,184,063	3.45
612: Animal producers	456,736	70,323	69,774 (99.2)	549 (0.8)	6,019,350	98,820	48,302,233	105.76
621: Forestry, Fishery and Aquaculture Workers	66,220	7770	7,676 (98.8)	94 (1.2)	172,213	16,920	189,133	2.86
631: Subsistence Crop Farmers	514,708	70,078	69,734 (99.5)	344 (0.5)	2,929,701	61,920	2,991,621	5.81
Total	13,273,520	1,785,847	1,704,655 (95.5)	81,192 (4.5)	36,868,427	14,614,560	51,482,987	3.88

*Note* Exchange rate: THB 32 = 1 US$ (Bank of Thailand)

The estimated average cost per farmer of all OIIs was $3.88USD (Table 4). If this cost were not absorbed by the UCHS, farmers would have had to spend an average of 0.2% of their annual income for medical costs to treat their OIIs. Animal producers suffer more expensive injuries compared to other farmers, largely from handling livestock. If their medical costs were not absorbed by the UCHS, they would have had to spend an average of 7% of their annual income for medical treatment of OIIs.

## Discussion

This is the first report on the number and medical treatment costs of occupational injuries and illnesses in the Thai agricultural sector. Other reports on OIIs have used data from the Workers Compensation System, which are only available for formal sector employees. This study is unique in using UCHS data to identify 1,704,655 occupational injuries among agricultural workers for 2017 (Table 1), different than the 17,481 agricultural injuries (16.9% of all injuries) reported to the National Injury Surveillance System of Thailand from 2002 to 2010 [25]. Clearly, Thailand sorely needs a robust system of OII data collection and surveillance for all informal sector workers.

### Occupational injuries in Thai agriculture

The ISCO sub-code of 6111 (Field, Crop, and Vegetable Growers) had the largest number of visits and medical costs for occupational injuries (Table 1). The risks of injury among small family farmers growing crops such as rice, sugarcane, fruits, vegetables, and other field crops, result from the wide range of activities and equipment used during land preparation, planting, cultivation, and harvesting [26, 27]. Animal producers and livestock handlers suffered more severe injuries, as evidenced by higher per visit medical costs for injuries (Table 1). Others have also identified animal handling for its high health and safety risks [28, 29].

### Occupational illnesses in Thai agriculture

Musculoskeletal Disorders (MSDs) have previously been reported as a common problem in Thai agriculture [26]. This, however, is the first study to estimate the annual medical costs attributed to MSDs (over $11.8 million), or about 28% of the total medical cost of all agricultural OIIs. In the U.S., MSD cases accounted for 31% of all worker injury and illness cases reported by U.S. Bureau of Labor Statistics [30].

Hearing loss has also been identified as a hazard in Thai agriculture, with a prevalence of up to 88% in some farming populations [31]. Exposures to ototoxicants such as pesticides may also play a role in the high prevalence of hearing loss [32]. The 15,182 cases identified in 2017 (Table 2) highlight the need for education about hearing protection and the provision of hearing protection devices to Thai farmers.

Although others have reported the problem of pesticide poisoning among those who mix or apply pesticides in Thai agriculture [8, 26], it appears that, like the imports of pesticides into Thailand [33], poisoning cases are increasing. In 2015, the estimated number of pesticide poisoning cases was 10, 177 [19] compared to the 13,995 cases we reported (Table 3). This is the first study to estimate the annual medical treatment costs attributable to pesticide poisoning (over a quarter of a million USD). We do not know the reason that the medical treatment costs for herbicide and fungicide poisoning are higher per case than for other pesticide poisoning cases. However, on June 1, 2020 the Thai government put in place bans on the use of the highly toxic herbicide paraquat and the insecticide chlorpyrifos and restricted the use of the herbicide glyphosate.

The UHCS rely on the primary care staff (most often a bachelor’s level nurse or public health officer) to identify occupational illnesses. The difficulty in diagnosing occupational illnesses without more extensive training may be reflected in the low number of occupational illness visits (3%) relative to injuries (97%) in our data. In addition, it is difficult to link occupational exposures to illness with a latency period between exposure and illness [25]. In the U.S., Occupational Safety and Health Act recordable occupational illnesses are also a small fraction of all OIIs and are believed to be under-reported [34].

### Addressing the Medical Costs of Treating occupational injury and illness (OIIs)

Establishing the cost associated with OIIs is essential for strategic planning by governments to enable them to allocate scare resources and put in place adequate prevention measures. The cost estimates for OIIs available for this paper are based solely on medical treatment costs.

In the economies of high resource countries in the West, the most important cost element for OIIs is not the medical treatment cost, but the immediate lost work time and productivity, plus the “lost return on investment in human capital” when a fatality or disability results in the inability to continue the job [35]. Such information is not available in Thailand for the informal sector, although some of it is available for the formal sector from the Workers Compensation system. Nevertheless, the medical cost estimates presented here do reflect a cost shifting of occupationally related healthcare costs to the national healthcare system.

### Prevention strategy

Thai farmers are currently covered under the Ministry of Labor’s Department of Labor Protection and Welfare “Guidance on Occupational Safety, Health and Environment for Informal Workers, 2013” [36]. Under this guidance, all informal workers, must promote safety and health at their own workplaces and meet all applicable standards. The government does not have a mechanism for the effective enforcement of this guidance nor a means to provide occupational safety and health support services to the informal sector. Thai regulations do not require the government agency or farmers to implement comprehensive OIIs prevention programs in agriculture.

Currently, the UHCS budget mainly covers medical treatment costs rather than prevention programs for OIIs. We recommend a re-allocation of a portion of the OII treatment costs enumerated here to providing agricultural workers with the critical components of prevention programs: hazard identification and remediation, training, and personal protective equipment. The Ministry of Public Health and the Ministry of Labor might develop safety programs that provide incentives for subsidizing the use of safely guarded machinery for farmers; training in sustainable and organic alternatives; subsidized personal protective equipment and additional training requirements before licensure for pesticide purchase. Outreach agricultural occupational safety and health training programs need to be set up around the country, in every district and sub-district, to reach agricultural workers in the remote rural areas. Prevention programs and subsidized using medical treatment funds could reduce OIIs and improve productivity.

### Limitations

Our study used Health Data Center data from 2017 resulting in an underestimate of current OII costs, as Universal Health Care System (UHCS) costs have increased over time. Due to the lack of adequate training and limited interest from the UHCS, many OIIs may not have been put into the proper Diagnostic Related Group. The determination that an illness was work related was not verified by the Bureau of Occupational and Environmental Disease and might be an over or under estimate. With regard to OII medical costs, our study assumed that the costs of medical treatment for most occupational illnesses were the same as the most frequent DRG category claim (DRG 04520: respiratory infection or inflammation without important cost and clinical complexity). Due to lack of data, these OII cost estimates represent only direct medical expenses. Because the number of days away from work or the number of days of job transfer or restriction is not available for informal workers, we do not include the other costs to workers and the economy due to daily lost productivity or income.

### Future direction

The U.S. Bureau of Labor Statistics conducts an annual Survey of Occupational Injuries and Illnesses that generates estimates of nonfatal workplace injury and illness rates. U.S. law requires agricultural businesses with more than 10 employees to participate in the surveys. This type of OII data collection process does not exist in Thailand and even in the U.S., information on OIIs in the informal sector is difficult to obtain. Therefore, in 1990, the U.S National Institute for Occupational Safety and Health developed the National Agricultural Workers Survey, a periodic surveillance program of nonfatal injuries to hired workers on U.S. crop farms, regardless of worker immigration status [37].

Thailand needs data collection tools and a database that can measure OII, lost income and workdays, and lost productivity for informal sector workers. It should be developed by the National Statistical Office of Thailand in collaboration with Ministry of Public Health and the Ministry of Labor. If collected through UCHS it will require sufficient training for healthcare personnel to classify an injury or illness as work related. It is important that family members who are injured on a family-owned farm, especially children, should be classified as having an OII. In addition, a method is needed to indicate that a family member was living on a family-owned farm, but not working at the time of exposure to a farm hazard that caused an injury or illness (pesticides, farm animals, farm tools) [38].

Alternatively, the Ministry of Agriculture or Ministry of Labor might develop a nationwide surveillance system to collect demographic, employment, and health and safety data in face-to-face interviews. Ideally, this would involve direct collection of data on working conditions, injuries and illnesses, and especially lost time, lost income and productivity, and related costs for the workers and their dependents. The timing of data collection should reflect the seasonality of agricultural production and employment. Better data on OIIs and the true costs, beyond medical treatment, including lost worktime and productivity, will provide incentives for changes in government policies to reduce UCHS expenditures and improve national agricultural productivity. Detailed information about the sources and nature of OIIs (crops, seasons, tools, activities) will aid efforts to target occupational health and safety training and interventions.

## References

[1] Office of National Economic and Social Development Council. National Income of Thailand 2016, chain volume measures. 2018. https://www.nesdc.go.th/main.php?filename=national_account. Accessed 10 Nov 2019.

[2] Office of National Economic and Social Development Council Economic Report, Thai Economic Performance in Q4 and 2018 and Outlook for 2019. 2019 https://www.nesdb.go.th/ewt_dl_link.php?nid=8660&filename=QGDP_report. Accessed 10 Oct 2019.

[3] Bank of Thailand. Key Economic Indicators Table 17: Population, Labour Force and Wage. 2018. https://www.bot.or.th/English/Statistics/Indicators/Pages/default.aspx. Accessed 10 Oct 2019.

[4] Pornpimol K, Noppanun N, Chalermchai C, Wisanti L, Susan W, Craig S (2015). Informal Workers in Thailand; Occupational Health and Social Security Disparities. New Solutions..

[5] Orawan K, Pornpimol K, Susan W (2015). Occupational health and safety in Thailand: gaps and recommendations, with a focus on pesticide. New Solut.

[6] Tangcharoensathien V, Witthayapipopsakul W, Panichkriangkrai W, Patcharanarumol W, Mills A. Health Systems Development in Thailand: a solid platform for successful implementation of Universal Health Coverage. The Lancet.com. 2018. https://www.thelancet.com/action/showPdf?pii=S0140–6736%2818%2930198–3. Accessed 5 July 2019. 10.1016/S0140-6736(18)30198-3 29397200

[7] Paek CS, Meemon N, Wan THT. Thailand’s Universal Coverage Scheme and its impact on health-seeking behavior. SpringerPlus. 2016 https://www.ncbi.nlm.nih.gov/pmc/articles/PMC5104696/pdf/40064_2016_Article_3665.pdf10.1186/s40064-016-3665-4. Accessed 5 July 2019. 10.1186/s40064-016-3665-4 PMC5104696 27933235

[8] International Labour Organization Fact sheet. Universal Health-care Coverage Scheme: Thailand, 2017 https://www.ilo.org/wcmsp5/groups/public/-dgreports/-integration/documents/publication/wcms_568679.pdf. Accessed 5 July 2019.

[9] Wagstaff A, Manachotphong, W. Universal health care and informal labor markets: the case of Thailand (English). Policy Research working paper; no. WPS 6116. Washington, DC: World Bank Group. 2012; https://documents.worldbank.org/curated/en/481061468120871217/Universal-health-care-and-informal-labor-markets-the-case-of-Thailand. Accessed 5 July 2019.

[10] National Health Security Office. National Health Security Fund Management Manual Fiscal Year 2020. 2019.www.nhso.go.th/files/userfiles/file/2020/หน่วยบริการ/คู่มือ/63_คู่มือบริหารกองทุน%252063.pdf. Accessed 5 Oct 2019.

[11] Aspalter C, Pribadi TK, Gauld R (2017). Health care systems in developing countries in Asia.

[12] International Labour Organization. Estimating the Economic Costs of Occupational Injuries and Illness in Developing Countries: Essential Information for Decision-Makers. Programme on Safety and Health at Work and Environment (SafeWork). Geneva. 2012 https://www.ilo.org/safework/info/publications/WCMS_207690/lang--en/index.htm. Accessed 5 Jul 2019.

[13] Khiaocharoen O, Pannarunothai S, Zungsontiporn C, Riewpaiboon A. Relative Weight for Thailand Diagnosis Related Group. Thai CaseMix Centre (TCMC). Health System Research Institute’s Affiliated Agency. 2017.https://www.tcmc.or.th/main/index.php/107-tdrg-v-6–1. Accessed 5 July 2019.

[14] Thai CaseMix Centre (TCMC). Health System Research Institute’s Affiliated Agency. List of DRG and Relative Weight in Thai DRG Version 6.1.National Institute of Health of Thailand. 2017. :https://www.tcmc.or.th/main/index.php/file-download/file/103-list-of-drg-and-relative-weight-in-thai-drg-version-6–1. Accessed 1 June 2019.

[15] Lebeau M, Duguay P. The costs of occupational injuries. A review of the literatures. Studies and Research Projects, R-787. The Institut de Recherche Robert Sauve’ en sante’ et en securite du travail (IRSST). 2013.http://www.irsst.qc.ca Accessed 10 March 2018.

[16] Leigh JP, McCurdy AS, Schenker B. Costs of Injuries in Agriculture. Public Health Reports. 2001. https://www.rand.org/pubs/testimonies/CT103.html. Accessed 10 May 2018. 10.1016/S0033-3549(04)50039-0 PMC1497330 12034913

[17] Leigh JP, Du J, McCurdy A (2014). An Estimation of the US Government’s Undercount of Nonfatal occupational injuries and illnesses in agriculture.. Ann Epidemiol..

[18] International Labour Organization. International Standard Classification of Occupations. Structure, group definitions and correspondence table. ISCO-08. Geneva. 2012. https://www.ilo.org/wcmsp5/groups/public/-dgreports/-dcomm/-publ/documents/publication/wcms_172572.pdf. Accessed 10 June 2018.

[19] Bureau of Occupational and Environmental Diseases. Center for Disease Control, Ministry of Public Health; 2015. Manual for Occupational Health Service of Public Health officer at agricultural Health Clinic, Vol 3 (In Thai). 2015http://envocc.ddc.moph.go.th/uploads/media/manual/2Un.pdf. Accessed 10 Jun 2018.

[20] National Health Security Office. National Health Security Fund Management Manual Fiscal Year 2020. http:// www.nhso.go.th/files/userfiles/file/2020/หน่วยบริการ/คู่มือ/63_คู่มือบริหารกองทุน%252063.pdf Accessed 10 Dec 2019.

[21] Bureau of Occupational and Environmental Diseases. Center for Disease Control, Ministry of Public Health; 2017. Occupational Diseases Monitoring System, 2015. https://envocc.ddc.moph.go.th/uploads/media/manual/manual_%2520Envocc%25202.pdf. Accessed 5 May 2019.

[22] Napasintuwong Orachos. Rice Economy of Thailand. Agricultural and Resource Economics Working Paper. No.2562/1 2019. Department of Agricultural and Resource Economics, Faculty of Economics, Kasetsart University. AgEconSearh. 2019. https://ageconsearch.umn.edu/record/284119/?ln=en. Accessed 5 Mar 2020.

[23] Bank of Thailand. Thailand’s Macro Economic Indicators. National Economic and Social Development Board (NESDB). 2019. https://www.bot.or.th/App/BTWS_STAT/statistics/BOTWEBSTAT.aspx?reportID=80&language=TH. Accessed 7 May 2020.

[24] Social Security Office Thailand. Annual Report: Worker Compensation Fund 2016. Ministry of Labor. 2017. https://www.sso.go.th/wpr/assets/upload/files_storage/sso_th/0c1643575f028d88f1f5a3c52cb5c023.pdf. Accessed 6 June 2019.

[25] Sangchom Siripanich, Meanpoung P, Sangchatip A. Trends and Characteristics of Occupational Injuries in Thailand, 2002–2010. Outbreak, Surveillance and investigation Reports. Bureau of Epidemiology, Department of Disease Control, Ministry of Public Health, Thailand. 2014. htttp://osijournal.net. file:///C:/Users/user/Downloads/36-1-68-1-10-20161118.pdf. Accessed 3 March 2019.

[26] Nankongnab N, Kongtip P, Tipayamongkholgul M, Bunngamchairat A, Sitthisak S, Woskie S. Difference in Accidents, Health Symptoms, and Ergonomic Problems between Conventional Farmers Using Pesticides and Organic Farmers. Journal of Agromedicine. 2020. https://www.ph.mahidol.ac.th/phep/Mat2_2019.pdf. Accessed 4 March 2020. 10.1080/1059924X.2019.1607793 PMC6815233 31025608

[27] Kongtip P, Nankongnab N, Mahaboonpeeti R, Bootsikeaw S, Batsungnoen K, Hanchenlaksh C, Tipayamongkholgul M, Woskie S. Difference among Thai Agricultural Workers’ Health, Working Condition and Pesticide Use by Farm Type. Annals of Work Exposures and Health. 2018. https://www.ph.mahidol.ac.th/phep/mat1_2018.pdf. Accessed 5 June 2019. 10.1093/annweh/wxx099 PMC6669355 29390118

[28] Jadhav R, Achutan C, Haynatzki G, Rajaram S, Rautiainen R. Review and meta-analysis of emerging risk factors for agricultural injury. J Agromed. 2016. https://www.researchgate.net/publication/304014104_Review_and_Meta-analysis_of_Emerging_Risk_Factors_for_Agricultural_injury_final_published_version. Accessed 9 June 2019. 10.1080/1059924X.2016.1179611 27088816

[29] Karttunen JP, Rautiainen RH. Characteristics of and risk factors for compensated occupational injury and disease claims in dairy farmers: a case-control study. J Agric Saf Health. 2013. https://www.ncbi.nlm.nih.gov/pubmed/24400423. Accessed 7 May 2019. 10.13031/jash.19.10172 24400423

[30] Bureau of Labor Statistics. News Release: Nonfatal Occupational Injuries and Illnesses Requiring Days Away From Work 2015 US Department of Labor. 2016. https://www.bls.gov/news.release/pdf/osh2.pdf. Accessed 10 June 2019.

[31] Suwanno S, Pongmuttasaya P, Jankhonkaen S, et al. Prevalence of Accident and Hearing loss of Machine Handing by Agricultural Workers in Responsible Area of The Office of Disease Prevention and Control 7th Ubonratchatani Province. The Office of Disease Prevention and Control 7th Ubonratchatani Province. 2008.

[32] Choochouy N, Kongtip P, Chantanakul S, Nankongnab N, Sujirarat D, Woskie S. Hearing loss in agricultural workers exposed to pesticides and noise. Ann Work Exposures Health. 2019.https://www.ph.mahidol.ac.th/phep/Dusit5_2019.pdf. Accessed 8 Feb 2020. 10.1093/annweh/wxz035 PMC7312224 31161207

[33] Office of Agricultural Economics. Agro economic Data. Thailand: Quantity and Values of Agricultural Pesticide Import Year 2015–2019 (In Thai). 2020. https://www.oae.go.th/view/1/. Accessed 5 May 2020.

[34] William JW. Monthly Labor Review. Examining the completeness of occupational injury and illness data: an update on current research*,* U.S. Bureau of Labor Statistics. 2014 https://www.bls.gov/opub/mlr/2014/article/pdf/examining-the-completeness-of-occupational-injury-and-illness-data-an-update-on-current-research.pdf 10.21916/mlr.2014.24. Accessed 17 May 2020.

[35] Weil D, Valuing the Economic Consequences of Work Injury and Illness: A Comparison of Methods and Findings. NIOSH Conference, “Integrating Social, Economic, and Health Services Research” Denver, CO, June 13–15, 1999. Harvard University. Cambridge, MA. 1999.

[36] Ministry of Labor. Notification entitled guidance on occupational safety, health and environment for informal workers, 2013. https://osh.labour.go.th/index.php?option=com_k2&view=itemlist&layout=category&task=category&id=35&Itemid=232&limitstart=8. Accessed 20 March 2020.

[37] Hernandez T, Gabbard S. Findings from the National Agricultural Workers Survey (NAWS) 2015–2016. A Demographic and Employment Profile of United States Farmworkers. JBS International. 2018. https://globalmigration.ucdavis.edu/sites/g/files/dgvnsk821/files/inline-files/naws_research_report_13jan2019.pdf. Accessed 5 March 2020.

[38] Arcury AT, Quant AS. Occupational and Environmental Health Risks in Farm Labor. Hum Organ. 1998. 10.17730/humo.57.3.m77667m3j2136178. 10.17730/humo.57.3.m77667m3j2136178 PMC6774661 31579291

